# LC-ESI-MS/MS Polyphenolic Profile and In Vitro Study of Cosmetic Potential of *Aerva lanata* (L.) Juss. Herb Extracts

**DOI:** 10.3390/molecules27041259

**Published:** 2022-02-13

**Authors:** Aleksandra Pieczykolan, Wioleta Pietrzak, Katarzyna Dos Santos Szewczyk, Urszula Gawlik-Dziki, Renata Nowak

**Affiliations:** 1 Department of Pharmaceutical Botany, Medical University, 1 Chodźki Street, 20-093 Lublin, Poland; aleksandraoleszek@umlub.pl (A.P.); wioleta.pietrzak@umlub.pl (W.P.); k.szewczyk@umlub.pl (K.D.S.S.); 2 Department of Biochemistry and Food Chemistry, University of Life Sciences, 8 Skromna Street, 20-704 Lublin, Poland; urszula.gawlik@up.lublin.pl

**Keywords:** *Aerva* extract, polpala, ASE, LC-ESI-MS/MS, MRM, phenolic compounds, tiliroside, ORAC, cosmetic potential

## Abstract

The aim of the present study was to investigate the phenolic composition and the biological properties of different *Aerva lanata* (L). Juss. herb extracts obtained with the use of accelerated solvent extraction (ASE), i.e., a green, ecological method, for cosmetic purposes. All samples exhibited high DPPH^•^ (9.17–119.85 mg TE/g) and ABTS^•+^ (9.90–107.58 mg TE/g) scavenging activity. The extracts exhibited considerable anti-lipoxygenase (EC_50_ between 1.14 mg/mL and 3.73 mg/mL) and anti-xanthine oxidase (EC_50_ between 1.28 mg/mL and 3.72 mg/mL) activities, moderate chelating activity (EC_50_ between 1.58 mg/mL and 5.30 mg/mL), and high antioxidant potential in the ORAC assay (0.36–3.84 mM TE/g). Changes in the polyphenol profile of the analysed samples depending on the solvent and temperature used for the extraction were determined with the liquid chromatography/electrospray mass spectrometry (LC-ESI-MS/MS) method. Twenty-one phenolic compounds, including flavonoids and phenolic acids, were detected and quantified. It was shown that tiliroside was one of the main phenolic metabolites in the *A. lanata* (L.) Juss. herb., which may suggest that this compound may be largely responsible for the observed anti-inflammatory activity of the extracts. In addition, the studied extracts exhibited promising skin-related (anti-tyrosinase, anti-elastase, anti-collagenase, and anti-hyaluronidase) activity. This study showed that *Aerva lanata* (L.) Juss. contains high amounts of phenolic compounds, including tiliroside, and has good skin-related activities. Therefore, the plant may be interesting as a novel source of bioactive agents for cosmetic industries.

## 1. Introduction

Plant-based natural products have grown in popularity over the years due to their enormous potential for health benefits, mainly as antioxidants, antiradicals, and pro-inflammatory enzyme inhibitors. It is well known that oxidative stress is responsible for the pathogenesis of chronic diseases and ageing [1]. In particular, ageing and ageing-related disorders are intrinsically related with redox imbalance and oxidative stress. Due to their remarkable potential in both the treatment and prevention of oxidative stress-related diseases, plants play a vital role as a source of biologically active natural products with pharmaceutical, cosmetic, and dermatological importance [2,3]. Such a broad range of the possible uses of plants in pro-health practice is possible due to their chemical composition and the presence of many groups of secondary metabolites with a wide spectrum of biological activity.

Among them, plant polyphenols are of particular interest. Polyphenols, including flavonoids and phenolic acids, are a large and structurally diverse group of natural bioactive substances. These compounds show diverse and multidirectional biological activity, especially strong antioxidant and antiradical activity. For this reason, polyphenolic extracts are highlighted because they have proven anti-ageing, antimicrobial, and solar photoprotection supporting activity, which is a very important feature of cosmetic ingredients [2].

*Aerva lanata* (L.) Juss. (AL; common name: polpala or mountain knotgrass) is a popular plant used in folk medicine. Due to its use in traditional medicine in the treatment of diabetes, arthritis, and urinary system diseases, the plant is of great interest to the pharmaceutical and nutraceutical industries [4]. *A. lanata* (L.) Juss. contains a rich composition of secondary metabolites, e.g., alkaloids, sterols, and phenolic compounds. For example, the literature provides information about the presence of some flavonoid compounds in polpala, e.g., kaempferol, myricetin, apigenin, chrysin, rutin, quercetin, and isorhamnetin [5,6,7]. However, there is still no comprehensive research on phenolic secondary metabolites in the herb of *A. lanata* (L.) Juss. [8].

Analysis of plant metabolites requires an efficient extraction method that provides a high extraction yield as well as a high specificity for the compounds of interest. In our previous study, we showed a multi-stage liquid/liquid extraction procedure of free and bound forms of phenolic acids from the herb of *A. lanata* (L.) Juss. to investigate their content, composition, and biological activity [9]. The method was very useful to obtain high concentrations of phenolic acids; however, the high solvent consumption and low extraction yield in this procedure were not satisfactory. This encouraged us to conduct further detailed research to identify the most efficient method for the extraction of polyphenolic compounds from *A. lanata* (L.) Juss. and to determine their presence and biological activity, especially in the context of their potential use in cosmetology.

Some literature data and our previous experience showed that ASE is a ‘green extraction’ and fully automated process; it is highly appreciated for its effectiveness and may be easily used for extraction of polyphenols [10,11,12]. Thus, in this study, a simple and efficient ASE method and conditions have been developed for the first time to obtain polyphenolic-rich AL extracts with high antioxidant and anti-inflammatory activity. A detailed qualitative and quantitative analysis of active polyphenolic secondary metabolites was performed using LC-ESI-MS/MS. The biological properties of the extracts were analysed by estimation of the antioxidant activity (with DPPH^•^, ABTS^•+^, ORAC, and metal-chelating activity tests), anti-inflammatory ability (lipoxygenase and xanthine oxidase inhibitory tests), and anti-ageing potential (anti-tyrosinase, anti-elastase, anti-collagenase, and anti-hyaluronidase activity).

As a result, the anti-ageing-related bioactivities of different extracts of *A. lanata* (L.) Juss. were described and the potential of this under-investigated plant in cosmetic applications was indicated for the first time.

## 2. Results and Discussion

As shown in some literature data, *Aerva lanata* (L.) Juss. is a phytochemical-rich plant [7,13,14]. Our previous research suggested that phenolic derivatives may be largely responsible for the observed biological activity of AL samples fractionated by liquid/liquid extraction. From a practical point of view, this makes it possible to use plant extracts to achieve health effects. Therefore, it is important to develop a modern, effective, simple, efficient, and green ASE method and conditions for obtaining polyphenolic-rich AL extracts with broad biological activity.

### 2.1. Optimisation of Accelerated Solvent Extraction Conditions

Available literature data prove that ASE is a suitable technique for the extraction of phenolic or/and flavonoid compounds [10,12,15,16,17]. Thus, the first step of our study was to analyse the phenolic contents of extracts obtained from *A. lanata* (L.) Juss. herb with the accelerated solvent extraction (ASE) technique using different concentrations of solvents (water, ethanol, and water–ethanol) and extraction temperatures (60, 80, 100, and 180 °C) in order to indicate the best method of extraction for this type of active secondary plant metabolite.

The basic ASE parameters (static time, flush volume, and sample loading weight and pressure) were established during a preliminary study. Ethanol and water in different proportions were selected as extraction solvents in this study (Table S1), as they are safer and less toxic [18]. The extraction temperature and solvent concentration were optimised. The extracts obtained from *A. lanata* (L.) Juss. herb showed substantial but varying amounts of total polyphenols (TPC) and flavonoids (TFC) as well as antioxidant properties depending on the extraction conditions. The efficiency of extraction, TPC and TFC contents, and antioxidant activity assessed in the samples with the DPPH^•^ and ABTS^•+^ method are shown in Table 1.

The extraction yields varied depending on the type and concentrations of solvents and the extraction temperatures. The highest extraction efficiency (Table 1) varied from 3.56 to 38.24% for the E100 (60 °C) and W (180 °C) samples, respectively. The best results were achieved for the water extracts (from 15.23 to 38.24%); good results were also obtained for the 50% ethanol–water extracts (from 10.15 to 31.22%). The use of concentrated ethanol yielded the lowest results (3.56 to 12.2%). The water and water–ethanol solvent systems ensured a higher yield of extraction than the 100% ethanol solvent used in the present study. The efficiency of extraction from *A. lanata* (L.) Juss. was largely influenced by the temperature: higher temperatures contributed to higher extraction yields in all samples. The highest (two- to three-fold) increase in the extraction efficiency was observed with the temperature increase in the range from 100 to 180 °C, regardless of the type of solvent used. This finding can be explained by the known scientific fact that increased extraction temperature reduces the viscosity of the solvent and increases its ability to wet the matrix and dissolve the desired analytes. The added thermal energy also assists in breaking analyte–matrix bonds and encourages analyte diffusion to the matrix surface [19,20].

As shown by the results, the highest total phenolic content, varying between 28.78 mg of GA/g DE (of dry extract) and 53.43 mg of GA/g DE, was achieved in samples extracted by the 50% ethanol–water solvent. The lowest results obtained for the water extracts ranged between 16.87 mg of GA/g DE and 22.88 mg of GA/g DE. The use of 100% and 80% ethanol yielded similar results ranging from 15.83 mg of GA/g DE to 45.20 mg of GA/g DE and from 15.91 mg of GA/g DE to 46.24 mg of GA/g DE, respectively. The yields of most of the phenolic compounds increased proportionally with the increasing extraction temperature, reaching maximum values at 180 °C (excluding water extracts).

**Figure 1:**
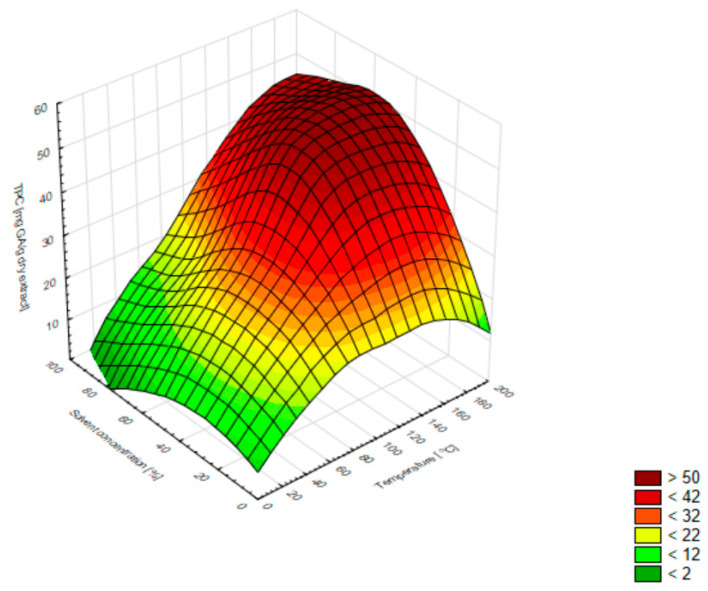
Three-dimensional surface chart of the total phenolic content vs. temperature and solvent concentrations for all extraction methods (smoothing of the smallest squares weighted by distance).

The 3D surface chart in Figure 1 shows that the TPC value increased as the extraction temperature increased. An increase in the concentration of the extractant to about 50% ethanol caused an increase in the TPC value; however, the higher ethanol concentrations did not exert a favourable influence on the phenolic content.

The effect of the temperature and solvent on the total flavonoid content was reflected in the following increase in TFC in the extract with the increase in the temperature in all samples: from 5.53 mg of Q/g DE in 60 °C to 11.54 mg of Q/g DE in 180 °C for the 100% ethanol concentration, from 6.53 mg of Q/g DE (60 °C) to 12.89 mg of Q/g DE (180 °C) for the 80% ethanol concentration, and from 6.79 mg of Q/g DE (60 °C) to 11.70 mg of Q/g DE (180 °C) for the 50% ethanol concentration.

**Figure 2:**
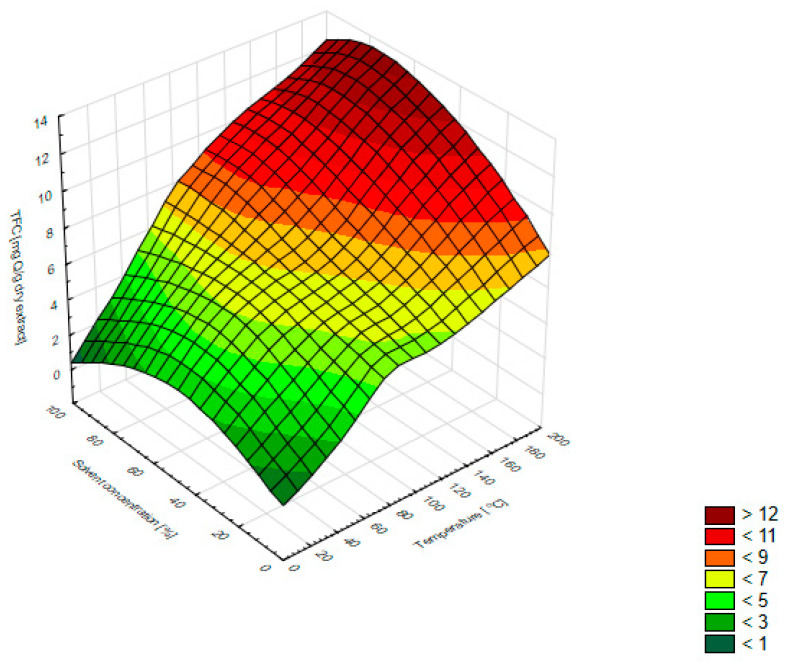
Three-dimensional surface chart of the total flavonoid content vs. temperature and solvent concentrations for all extraction methods (smoothing of the smallest squares weighted by distance).

Figure 2 shows the relationship between TFC, temperature, and solvent concentrations. The increase in the extraction temperature was accompanied by an increase in the TFC value. This was also confirmed by the calculated Pearson correlation coefficient of 0.835 (presented in Table 2). An increase in the concentration of the solvent to about 80% caused an increase in the TFC value; however, the higher ethanol concentration reduced the content of flavonoids.

There are numerous methods available for the evaluation of antioxidant capacity in plant extracts, but there is no standardised method for measuring the antioxidant capacity of all samples accurately. Therefore, we decided to determine the antioxidant capacity of extracts obtained from *A. lanata* (L.) Juss. with the common DPPH^•^ and ABTS^•+^ antiradical assays. These frequently used assays are fast, economical, and reliable.

**Figure 3:**
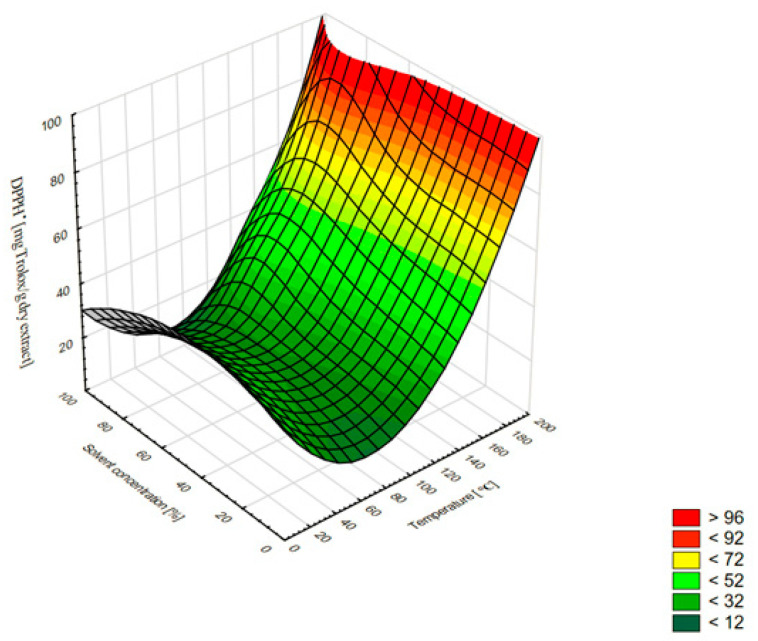
Three-dimensional surface chart of the DPPH^•^ vs. temperature and solvent concentrations for all extraction methods (smoothing of the smallest squares weighted by distance).

**Figure 4:**
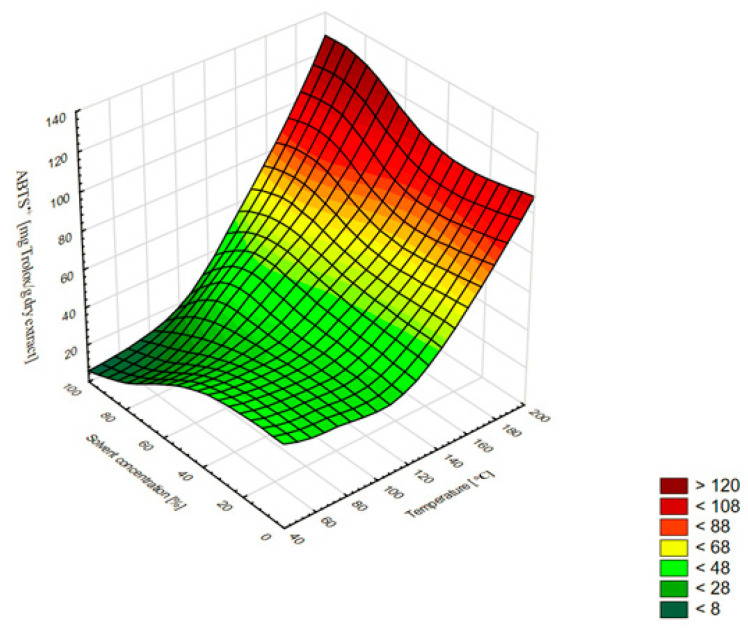
Three-dimensional surface chart of the ABTS^•+^ vs. temperature and solvent concentrations for all extraction methods (smoothing of the smallest squares weighted by distance).

The results show that the antioxidant capacities of *A. lanata* (L.) Juss. extracts were correlated with the temperature increase. The ABTS^•+^ radical scavenging effect and the DPPH^•^ radical scavenging activity of the different extracts exhibited a similar trend: their activity increased with the rise in the temperature. The 80% ethanol extract (180 °C) exhibited the highest DPPH^•^ (119.85 mg of Trolox/g DE) and ABTS^•+^ (107.58 mg of Trolox/g DE) scavenging activity. The lowest antioxidant properties in both tests were obtained when 100% ethanol was used as a solvent: 9.17 mg of Trolox/g DE at 80 °C (DPPH^•^) and 9.90 mg of Trolox/g DE at 60 °C (ABTS^•+^). The use of temperature up to 100 °C slightly improved the antioxidant properties, but the increase to 180 °C improved these properties several times. Similar relationships during the extraction of plant substances with the ASE method were observed by other authors [21,22]. In our study, the temperature was an important factor affecting not only the extraction efficiency and the composition of phenolic compounds, but also the antioxidant activity in both the DPPH^•^ and ABTS^•+^ radical cation methods. This was also confirmed by the high calculated Pearson correlation coefficients, i.e., 0.935 and 0.949 for DPPH^•^ and ABTS^•+^, respectively (Table 2). The optimal concentration of ethanol used for the extraction was about 50%. The use of the higher ethanol concentrations (>50%) caused a significantly greater decrease in antioxidant activity than the lower concentrations (<50%) (Figure 3 and Figure 4).

Satisfactory results for the total contents of phenolic and flavonoid compounds and antioxidant activity were obtained for the aqueous extracts, especially at the higher temperatures.

Our results for the contents of polyphenols and flavonoids in the AL herb are comparable to data reported previously by other authors [23,24,25]. There are some literature data about the antioxidant activity of polar extracts from *A. lanata* (L.) Juss. as well [23,24]. However, due to the differences in the methods and the mode of expression of the results, it is difficult to compare these data with ours.

### 2.2. Statistical Analysis

#### 2.2.1. Pearson’s Correlation Coefficients

To explain in detail the relationships between the antioxidant properties (in DPPH^•^ and ABTS^•+^ tests) of *A. lanata* (L.) Juss. herb extracts and the phenolic contents (TPC and TFC), correlations between the different extracting conditions were calculated (Table 2). It can be concluded that TFC was the main contributor to the DPPH^•^ and ABTS^•+^ free radical scavenging capacity of *A. lanata* (L.) Juss. (*r* = 0.880 and 0.816, respectively), whereas TPC exhibited a slightly lower but still high correlation (*r* = 0.720 and 0.722, for DPPH^•^ and ABTS^•+^, respectively).

As mentioned before, the temperature in the ASE method exerted a positive effect on the efficiency of extraction (*r* = 0.761), TPC (*r* = 0.694), TFC (*r* = 0.835), DPPH^•^ (*r* = 0.935), and ABTS^•+^ (*r* = 0.949), which means that high temperature is the most important factor for the acquisition of polyphenol- and flavonoid-rich *A. lanata* (L.) Juss. herb extracts with high antioxidant activity.

#### 2.2.2. Clustering Analysis

**Figure 5:**
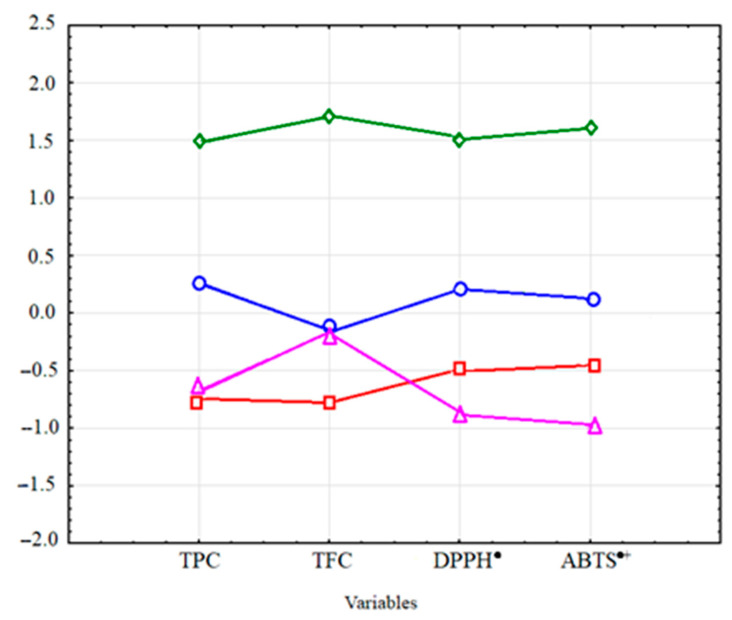
Graph of mean values for four clusters. Abbreviations: O—Cluster 1; □—Cluster 2; ◊—Cluster 3; Δ—Cluster 4.

The analysis of clusters using the k-means method indicated division of the obtained spectrophotometric results of the total contents of polyphenols, flavonoids, and antioxidant activities in the extracts into four clusters (Table 3, Figure 5).

The cluster analysis applied to the AL extracts facilitated the identification of the most and least efficient groups of conditions of the extraction methods. In this case, the k-means cluster analysis clearly demonstrated the best properties of procedures in Cluster 3, which comprised ethanol and ethanol–water extracts obtained at a temperature of 180 °C. The methods included in Cluster 3 ensured the highest total content of TPC and TFC, and the highest values of DPPH^•^ and ABTS^•+^, thus indicating the greatest antioxidant activity. As shown by the results, the concentration of the extraction solvent used at the very high temperature (180 °C) did not significantly affect the TPC, TFC, and antioxidant activity.

Satisfactory results were also obtained for Cluster 1. This set includes extracts obtained using 50% ethanol and temperatures of 60, 80, and 100 °C, the 80% ethanol extract at 100 °C, and the aqueous extract obtained at the extraction temperature of 180 °C. The results showed that the 50% concentration of ethanol used for extraction, regardless of the extraction temperature, yielded high results for TPC, TFC, and antioxidant activity. In addition, Cluster 1 includes the extract obtained with 80% ethanol at high temperature and the aqueous extract obtained at the highest temperature used.

Cluster 2 includes water extracts obtained at 60, 80, and 100 °C and ethanol extracts (80%) obtained at 60 and 80 °C. Finally, low values of TPC, TFC, and antioxidant activity were obtained in Cluster 4, which includes extracts obtained using 100% ethanol and temperatures of 60, 80, and 100 °C. The weakest solvent was 100% ethanol, and the increase in temperature in this variant did not affect the results significantly.

It is worth noting that our study is the first to report the content of phenolic compounds in ASE extracts from AL herb and the influence of different extraction conditions on their yield. Additionally, it was indicated that ASE extraction, especially at the higher temperatures, was able to release very high amounts of antioxidant active polyphenols from this plant material. This data may be of great importance for the potential commercial use of AL as a source of biologically active phenolic compounds.

### 2.3. Polyphenolic Composition and Biological Activities of Selected A. lanata (L.) Juss. Extracts

The statistical analysis (Section 2.2) showed a decisive influence of the temperature on the composition and antioxidant activity of the obtained extracts from *A. lanata* (L.) Juss. Hence, it was justified to conduct a detailed analysis of the polyphenol composition and biological activity tests for selected samples obtained in the extreme temperature conditions and different solvent (ethanol: water) compositions.

#### 2.3.1. LC-ESI-MS/MS-MRM Analysis of Polyphenols

The qualitative and quantitative analysis of phenolic compounds in the analysed samples was performed using the liquid chromatography-electrospray ionisation-tandem mass spectrometry method (LC-ESI-MS/MS) in multiple reaction monitoring mode (MRM). As a result, the occurrence and content of 10 phenolic acids and 11 flavonoids (Table 4, Tables S2 and S3) was revealed.

The data presented in Table 4 show that 4-hydroxybenzoic acid was the dominant phenolic acid (9.1 to 521.0 μg/g DE) in AL extracts obtained at 60 °C, accounting for several times higher quantities than at 180 °C in the case of all solvents. A higher level of this compound was observed in the water samples as well. Another phenolic acid present in a substantial amount in all extracts was *p*-coumaric acid (86.3–157.5 μg/g DE), especially in the 50 and 80% ethanol–water extracts and at the lower temperatures. It was observed that the lower temperatures and the mixture of water and ethanol as a solvent increased the content of this compound obtained from the *A. lanata* (L.) Juss. Herb extracts. In the 100% ethanol and extracts, better results were shown for the high temperature. Vanillic acid was present only in the 100% ethanol and 60 °C variant of extraction (25.3 μg/g DE). Gentistic, gallic, caffeic, and ferulic acids were detected in trace amounts or were not detected, depending on the sample. In turn, salicylic acid was detected only in trace amounts.

*p*-Coumaric acid was present in large amounts in water samples obtained at 180 °C, but was undetectable in water extracts at the low temperature. This finding may be connected with the results of our previous study and may indicate the possibility of the release of this compound from its bound forms at high temperature in water as a solvent. Our previous research showed that coumaric acid was present mainly in a bound form, mainly as an ester, a glycoside, or a glycoside-ester [9].

In the case of flavonoids, our results showed the highest content of tiliroside in all ethanol and water–ethanol AL extracts. However, this compound occurred at a similar level in extracts obtained with 100% ethanol, regardless of the temperature used. In turn, a 6–7-fold decrease in its content was observed in the water–ethanol extracts at high temperature. The highest amount of tiliroside was detected in the 80% ethanol extract at 60 °C (1565.0 µg/g DE). These results suggest that AL is a very good source of tiliroside. Based on these results, it can be concluded that the use of ASE with a water–ethanol solvent at moderate temperatures is a good method for the extraction of this compound from plant material. It is worth emphasising that the most abundant flavonoid constituent in the obtained AL extracts, i.e., tiliroside, is a kaempferol 3-*O*-β-D-(6″-*O*-(*E*)-*p*-coumaroyl)glucopyranoside that is famous for its high and multidirectional pharmacological activity. The compound exhibits unquestionable antioxidant, free-radical scavenging, and anti-inflammatory properties [26]. Given these activities, tiliroside is used in the cosmetics industry (it is also included in the International Nomenclature of Cosmetic Ingredients list), mainly as an ingredient in cosmetics that soothe skin redness and itching [27]. Takeda et al. showed that tiliroside improved the functioning of the skin barrier and retained moisture in skin conditions with ceramide deficiency associated with the use of surfactants and atopic dermatitis [28]. Tiliroside activated proteasome in normal human fibroblasts and delayed cell ageing, indicating that it may have potential anti-ageing activity [29]. It also inhibited intracellular tyrosinase activity and melanin production. This supports the idea that this compound can be a potential skin-whitening agent in cosmetics [30]. Moreover, many patents have shown that the compound is used in cosmetics [31,32,33].

*Aerva lanata* (L.) Juss. is traditionally applied to the skin as an astringent and emollient agent [34]. The plant is used for healing wounds and in the treatment of skin ailments, which was confirmed by Devi et al. [35]. However, the scientific basis of the claims of its cosmetic use is rudimentary and should be further elucidated.

Additionally, high contents of astragalin (kaempferol 3-O-glucoside) were obtained in our study as well (82.0–297.0 µg/g DE). Narcissin (isorhamnetin-3-O-rutinoside) was less abundant (26.4–118.0 µg/g DE), but it was present in all samples. The highest results of tiliroside, astragalin, and narcissin were obtained at low temperature (60 °C) and with the use of the water–ethanol solvents (50 or 80% ethanol).

The highest amounts of the quercetin flavonoid aglycone were found in 50% ethanol at 180 °C (316.0 µg/g DE). At low temperature, it was only present in the 80% ethanol sample, and its level in this variant was 10-fold lower than at high temperature. Isorhamnetin was present in larger quantities at 180 °C as well. Prunetin was present in all samples, and its highest amounts were obtained with 80% ethanol. Its content in the extracts prepared with different temperatures did not differ greatly, indicating that the extraction temperature did not have a significant impact on its recovery from the plant material. Rhamnetin was detected in trace amounts. It was observed that water was not a good extractant for this class of compounds from the *A. lanata* (L.) Juss. herb. However, the higher temperatures and the water–ethanol solvents increased the elution of rhamnetin.

#### 2.3.2. Oxygen Radical Absorbance Capacity (ORAC)

Antioxidants also play an important role in the treatment of diseases with inflammation [36]. ORAC measures the peroxyl radical scavenging capacity by monitoring the degree of inhibition of peroxyl radical-induced oxidation [37]. Samples obtained at 60 °C showed noticeably lower activity (0.358–0.808 mM Trolox/g DE). The strongest antioxidant effect was detected in all extracts prepared at 180 °C (1.852–3.840 mM Trolox/g DE). The highest value was determined in the extract obtained in 50% ethanol at 180 °C. All solvent systems showed increasing ORAC activity with the increasing ASE temperatures, which may be explained by the combined effects of increased mass transfer and analyte solubility as well as reduced analyte–matrix bonding. The ORAC activity levels increased 5.2-, 2.5-, 4.3-, and 2.9-fold in the 100% ethanol, 80% ethanol, 50% ethanol, and water extracts, respectively. The water extracts showed slightly lower activity than the 50% and 80% ethanol solvents (Table 5).

#### 2.3.3. Chelating Power (CHEL)

Since the antioxidant potential is also related to ion chelating capacity, we decided to determine the chelating power of eight selected AL extracts. The examined extracts were characterised by quite moderate metal-chelating ability (Table 5). The ethanol extract had higher activity than the AL water–ethanol samples. The best result was achieved using a low temperature and the 100% ethanol concentration (EC_50_ = 1.58 mg/mL DE). In all samples, the worst results were obtained using higher temperatures.

#### 2.3.4. Pro-Oxidant Enzyme Activity

Oxidative stress is involved in the pathogenesis of many diseases including inflammatory diseases. Overproduction of reactive oxygen species (ROS) leads to inflammation by stimulation of cytokines and activation of pro-inflammatory enzymes, e.g., lipoxygenase, xanthine oxidase, hyaluronidase, and inducible nitric oxide synthase. Lipoxygenases (LOX) are capable of generating such mediators as leukotrienes and prostaglandins, which can provoke several inflammatory diseases. In turn, xanthine oxidases (XO) play a role in metabolic disease, namely gout, which is closely associated with inflammation. Pro-inflammatory enzymes play an important role in the pathogenesis of inflammation; therefore, inhibition of these enzymes is considered as a target for the management of diseases associated with oxidative stress and inflammation [36]. As presented in Table 6, the highest LOX inhibitory potential was found for the water extracts at the temperature of 60 °C and 180 °C (EC_50_ = 1.14 and 1.15 mg/mL DE, respectively) and the 50% ethanol extract at 180 °C (EC_50_ = 1.24 mg/mL). In turn, the lowest values were determined in the case of the 100% ethanol extracts at the temperature of 60 °C and 180 °C (EC_50_ = 3.73 and 3.65 mg/mL, respectively) and the 80% ethanol extract at 60 °C, where the EC_50_ was 3.51 mg/mL. Overall, the solvent type has a major influence on the activity of *A. lanata* (L.) Juss. extracts against lipoxygenase.

**Figure 6:**
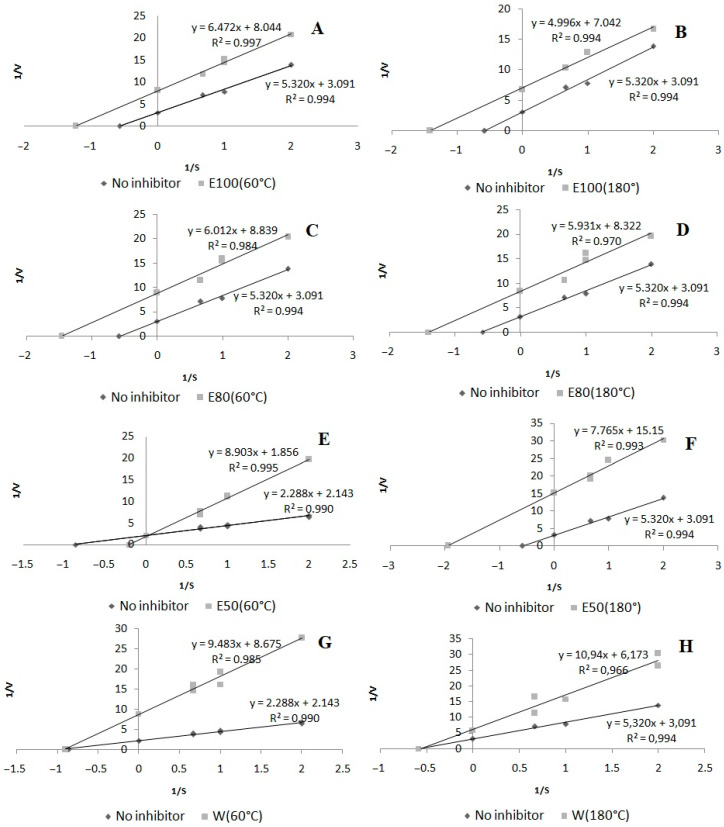
Mode of lipoxygenase inhibition by extracts from *Aerva lanata* (L.) Juss.: (**A**) 100% ethanol/60 °C; (**B**) 100% ethanol/180 °C; (**C**) 80% ethanol/60 °C; (**D**) 80% ethanol/180 °C; (**E**) 50% ethanol/60 °C; (**F**) 50% ethanol/180 °C; (**G**) water/60 °C; (**H**) water/180 °C.

As shown in Figure 6G,H, a non-competitive type of LOX inhibition was obtained in both water extracts with the highest activity. A competitive mode of inhibition was noted in the case of the 50% ethanol extract at 60 °C (Figure 6E). The other tested extracts exhibited an uncompetitive mode of action.

**Figure 7:**
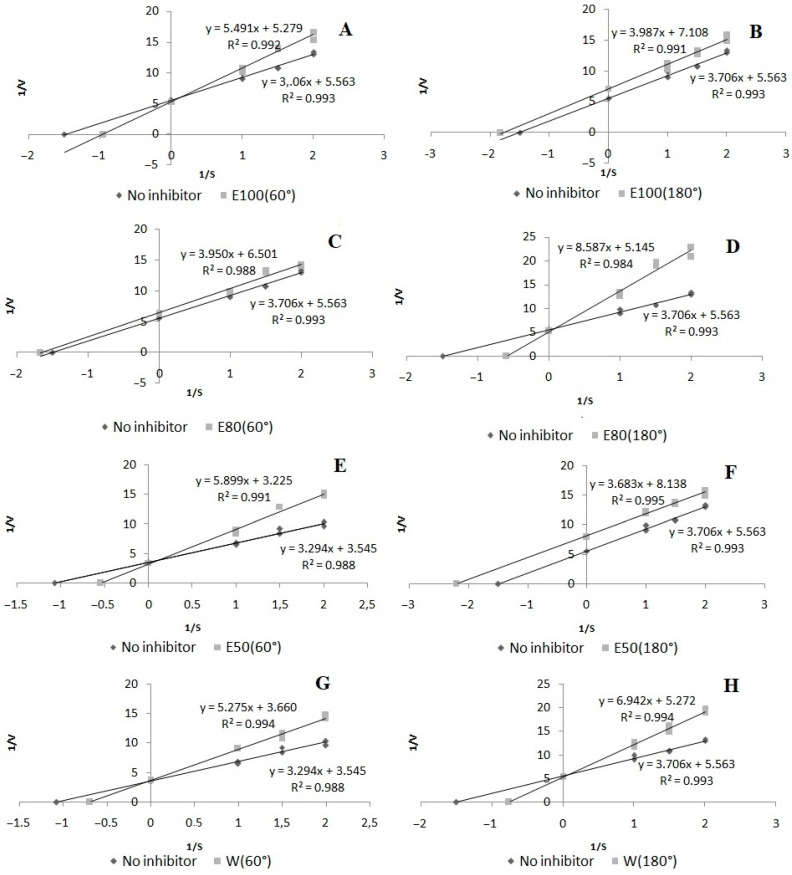
Mode of xanthine oxidase inhibition by extracts from *Aerva lanata* (L.) Juss.: (**A**) 100% ethanol/60 °C; (**B**) 100% ethanol/180 °C; (**C**) 80% ethanol/60 °C; (**D**) 80% ethanol/180 °C; (**E**) 50% ethanol/60 °C; (**F**) 50% ethanol/180 °C; (**G**) water/60 °C; (**H**) water/180 °C.

In the case of XO inhibition, the highest potential was found for the 50% ethanol extract at 180 °C (EC_50_ = 1.28 mg/mL DE), whereas the lowest values were determined in the case of the water extract at 60 °C and 100% ethanol at 60 °C (EC_50_ = 3.48 and 3.32 mg/mL, respectively). The use of water–ethanol solvents at higher temperatures significantly improved the activity of the extracts against xanthine oxidase. The extracts showed different modes of XO inhibition. The 80% ethanol extract at 60 °C and the 50% and 100% ethanol extracts at 180 °C acted as uncompetitive inhibitors, while the water extract at 60 °C exhibited a non-competitive mode of inhibitory action. The other extracts showed a competitive mode of action (Figure 7).

### 2.4. Statistical Correlations between the Biological Activities and the Content of Phenolic Compounds in Selected A. lanata (L.) Juss. Extracts

In scientific research, plant phenolic compounds (flavonoids, phenolic acids, etc.) have been an endless source in terms of the isolation of active compounds from plant extracts, the assessment of their chemical structure, and the characterisation of their wide spectrum of biological properties. Many studies were undertaken to show the influence of phenolic compounds on the biological activity of plant extracts [38]. Similarly, Pearson’s correlation coefficients (*r*) between the amounts of phenolic acids and flavonoids determined with the LC/MS method and the extraction conditions and biological activities were calculated in our study for selected samples (Table 6).

The 4-hydroxybenzoic acid (*r* = −0.928) and tiliroside (*r* = −0.536) compounds were shown to be sensitive to high temperatures, which reduced their contents. A beneficial effect of the temperature increase on the quantitative composition was observed in the case of quercetin (*r* = 0.760), isorhamnetin (*r* = 0.737), and kaempferol (*r* = 0.620). The greatest influence of the increase in the solvent concentration on the content of the tested compounds was observed in the case of astragalin (*r* = 0.861), narcissin (*r* = 0.646), and protocatechuic acid (*r* = 0.636).

Among all active compounds analysed in our study, special attention should be paid to flavonoid aglycones, mainly quercetin, kaempferol, and isorhamnetin, whose highest contents were determined in samples with the highest activity in the DPPH^•^, ABTS^•+^, ORAC, and XO in vitro tests. The values of Pearson’s coefficients suggest their role in the increase in the antioxidant activity and the XO inhibition ability of the analysed samples. Quercetin and isorhamnetin had a high influence on antioxidant activities (*r* > 0.7) and XO inhibition (*r* > −0.6), and the similar action of these compounds may be related to the fact that isorhamnetin is a metabolite of quercetin [39]. In turn, kaempferol exhibited a moderate correlation with antioxidant activities (*r* > 0.5) and XO inhibition (*r* > −0.5). As shown in other studies, quercetin is a strong inhibitor of XO [40]. These relationships were confirmed in our study as well (Table 6).

The results presented above are in agreement with the fact that it is mainly flavonoids that are correlated with the antioxidant activity reported in the literature. Although the correlations of the activity with some of the compounds (kaempferol, quercetin, and isorhamnetin) presented in this paper are significant (Table 7), lower or insignificant correlations were calculated for the other compounds. Additionally, the extracts were found to have high LOX inhibition activity but low correlations with the content of the analysed compounds. It is therefore reasonable to conclude that, in addition to phenolic compounds, other molecules contribute to anti-inflammatory activities.

### 2.5. Skin Ageing-Related Enzyme Activity

Skin plays an essential role as a barrier protecting internal organs against physical, chemical, and biological factors. Excess oxidative stress is harmful and results in unhealthy and ageing skin with wrinkles, dryness, elasticity loss, and uneven pigmentations. In addition, elevated levels of ROS can cause not only senescence but also other human diseases. Antioxidants provide a great defence against ROS and reduce oxidative stress. Compounds with strong antioxidant activity additionally facilitate skin protection against oxidative damage along with delaying the skin ageing process [41].

Therefore, based on all the results, in particular the antioxidant and anti-inflammatory activity as well as the high content of tiliroside in the obtained samples, we analysed enzymes related to the skin. It should be emphasised that the presented results are preliminary and it is necessary to conduct detailed analyses, taking into account the mechanism of action of the compounds. The present study consisted in the screening of selected *Aerva lanata* (L.) Juss. extracts (water, water–ethanol, and ethanol) for the content of quercetin, tiliroside, kojic acid, and epigallocatechin gallate and their in vitro inhibition of skin ageing-related enzymes, namely tyrosinase, collagenase, elastase, and hyaluronidase (Table 8).

Melanin is a major component of the skin, eye colour, and hair. Overproduction of melanin may cause skin disorders, including freckles, melasma, age spots, senile lentigines, and post-inflammatory hyperpigmentation leading to flaws and premature ageing in appearance [41]. The tyrosinase enzyme is a key enzyme responsible for the production of melanin and the rate-limiting step during melanin pigmentation. Kojic acid is a whitening material inhibiting the activity of the tyrosinase enzyme. However, skin irritation was reported after application thereof [42].

Fibroblasts and proteins, such as collagen and elastin, are the outermost parts of the skin (ECM). Collagen is the most abundant protein structure in the human dermis layer providing the tensile strength of the skin; in turn, elastin, i.e., a fibre network located in the connective tissue, is responsible for the elastic recoil property. These proteins are necessary to the skin, as they play a major role for plumpness, flexibility, integrity, and elasticity, keeping skin youthful and healthy. ROS accumulated after skin exposure to photoageing stressors can indirectly activate dermal enzymes such as collagenase and elastase, which degrade collagen and elastin, respectively [41].

Hyaluronic acid (hyaluronan) can be commonly found in the dermal compartment of skin and the epidermal layer. Hyaluronic acid mainly promotes skin rejuvenation, contains moisture, increases viscosity, and reduces the permeability of extracellular fluid. Hyaluronidase is an hyaluronan destructive enzyme leading to loss of strength, flexibility, and moisture and, subsequently, skin ageing [41].

Many studies are being conducted to discover new effective plant materials with broad biological activity. In recent decades, the awareness of skin ageing has become one of the most highlighted issues for scientists, and the numbers of skin ageing studies are still increasing. The inhibitory effects (mainly anti-tyrosinase, anti-collagenase, anti-elastase, and anti-hyaluronidase activities) of *Aerva lanata* (L.) Juss. extracts, quercetin, tiliroside, kojic acid, and EGCG assessed in this study are summarised in Table 7.

The different types of skin ageing-related enzymes have important roles in photoageing, and their activities are increased in inflammation. Two of the tested extracts exerted an extremely good tyrosinase inhibitory effect comparable to the standard, i.e., kojic acid in the extract obtained using the 80% ethanol concentration at 60 °C (42.32 µg/mL ± 0.57), followed by the 50% ethanol concentration at 60 °C (46.08 µg/mL ± 0.29). In addition, the activity of tiliroside was comparable (24.92 µg/mL ± 0.29) to that of kojic acid (28.42 µg/mL ± 0.11). Based on the present results and the content of active compounds in AL, it is assumed that tiliroside is responsible for the inhibition of the activity of the tyrosinase enzyme. Another study confirmed that tiliroside is a natural tyrosinase inhibitor that is suitable for cosmetic use [30].

Furthermore, it was noted that the highest collagenase and elastase inhibitory effect was exhibited by the variants with the 50% ethanol concentration at 180 °C (21.76 µg/mL  ±  1.27 and 35.81 µg/mL ± 0.81, respectively) and the 80% ethanol concentration at 180 °C (59.73 µg/mL  ±  0.31 and 22.54 µg/mL  ±  1.86, respectively). In addition to these extracts, another AL extract exhibited fairly high activities against both collagenase and elastase, with values of 78.47 µg/mL ± 0.29 and 57.26 µg/mL ± 0.30 in the 100% ethanol concentration at 180 °C. The lowest activity was observed for the water extracts. In our study, the extracts showed no significant difference in anti-hyaluronidase activity. The extracts obtained at the 80% ethanol concentration and 60 °C exhibited the best hyaluronidase inhibition activity (117.54 µg/mL ± 4.82), compared to the other variants. Most importantly, such activity was comparable with that of the positive standard—EGCG (94.85 µg/mL ± 0.52). In turn, the hyaluronidase inhibition activity of the extracts obtained at 180 °C was lower. The use of lower temperatures was associated with higher anti-hyaluronidase activity.

Pearson’s correlation coefficients were calculated between the amounts of the determined compounds and the tyrosinase, collagenase, elastase, and hyaluronidase activities (Table 8). The anti-tyrosinase activity was found to be significantly correlated with the sum of flavonoids (with particular emphasis on flavonoid glycosides; *r* = −0.731). Additionally, our results showed that the anti-collagenase and anti-elastase activities were correlated with the content of flavonoid aglycones (*r* = −0.623 and −0.939, respectively). In the case of the anti-hyaluronidase activity, the sum of phenolic acids was observed to exert a considerable impact (*r* = −0.610). 

## 3. Materials and Methods

### 3.1. Chemicals

Methanol, ethanol, acetic acid, FeCl_2_, Na_2_EDTA, and sodium carbonate (anhydrous) were purchased from Avantor Performance Materials Poland (Gliwice, Poland). Trolox, gallic acid, 2,2′-azino-bis-3(ethylbenzthiazoline-6-sulphonic acid) (ABTS^•+^), 2,2-diphenyl-1-picrylhydrazyl (DPPH^•^), collagenase from *Clostridium histolyticum*, elastase from porcine pancreas, hyaluronic acid, Bovine Serum Albumin, sodium phosphate monobasic solution, sodium phosphate dibasic solution, sodium chloride solution, sodium acetate, acetic acid, (-)-epigallocatechin gallate (EGCG), tyrosinase from mushrooms (≥1000 unit/mg solid), Levodopa (L-DOPA), N-[3-(2-Furyl)acryloyl]-Leu-Gly-Pro-Ala (FALGPA), N-Succinyl-Ala-Ala-Ala-p-nitroanilide (SANA), Tricine (≥99%; titration), Folin–Ciocalteu reagent, 2,2′-azobis (2-methylpropionamide) dihydrochloride (AAPH), ferrozine, aluminium chloride, soybean 15-lipoxygenase, xanthine oxidase, linoleic acid, allopurinol, caffeic, *p*-coumaric, gallic, ferulic, protocatechuic, 4-hydroxybenzoic, vanillic, syringic, salicylic, and rosmarinic acids, rutin, kaempferol, luteolin, and LC-MS-grade acetonitrile were obtained from Sigma-Aldrich Chemical Co. (St. Louis, MO, USA). Catechin, luteolin-7-O-glucoside, luteolin 3′,7′-diglucoside, naringenin 7-O-glucoside, gentisic, and sinapic acid were purchased from ChromaDex (Irvine, CA, USA). Astragalin, apigenin, tiliroside, and fluorescein sodium salt were supplied by Roth (Karlsruhe, Germany), and quercetin was purchased from Fluka (Buchs, Switzerland). Phosphate-buffered saline (PBS) was purchased from Gibco (Carlsbad, CA, USA). All the chemicals were of analytical grade. LC-grade methanol and formic acid were purchased from J.T. Baker (Phillipsburg, USA). LC-grade water was prepared using a Millipore Direct-Q3 purification system (Bedford, MA, USA).

### 3.2. Plant Material

Herb of *Aerva lanata* (L.) Juss. (series no. 51,018) was purchased from the herb company “Ликтpaви” (Kyivs’ke Hwy, 21, Zhytomyr, Zhytomyr Oblast, Ukraine). According to the information provided by the manufacturer, the herb material was properly dried and packed. The analysis and tests were conducted before the expiration date, which was specified as October 2021. In order to standardise the tested samples, 20 packages of *A. lanata* (L.) Juss. herb were blended together. The portions were mixed by shaking vigorously for 5 min and placed together in a paper bag. The obtained herb blend was then stored in a sealed paper bag in a cool dry place and protected from direct light.

### 3.3. Preparation of Plant Extracts

A fully automated ASE 150 system (Dionex Corporation, Sunnyvale, CA, USA) was used to perform the ASE process and sixteen types of extracts were obtained. The pulverised *Aerva lanata* (L.) Juss. herb (2 g) was mixed with diatomaceous earth and loaded into a stainless-steel cell. The extraction cell was placed into the carrousel and submitted to the experimental procedure at 60 °C, 80 °C, 100 °C, and 180 °C with water and different ethanol concentrations (50%, 80%, and 100%) as solvents in three cycles for 10 min each. The solvent was purged from the cell with nitrogen, and depressurisation took place. All extracts were prepared in triplicate. Eluates were combined and filled to 50 mL with the extraction solvent to unify the sample volume and concentration. The extracts were filtered, evaporated to dryness under vacuum (Heidolph Basis Hei-VAP Value evaporator, Schwabach, Germany), lyophilised in the Free Zone 1 apparatus (Labconco, Kansas City, KS, USA), weighed, and kept at −18 °C prior to analysis. Then, each sample was carefully redissolved in the same solvent as that used for extraction to obtain stock solutions at suitable concentrations, and filtered through a 0.45 μm membrane filter (Agilent Technologies, USA) prior to the analyses of active components.

The extracts were marked with symbols denoting the type of solvent (E: ethanol; W: water) and the concentration of ethanol (100: 100%, etc.); additionally, the extraction temperatures were provided in brackets (60 °C: 60 °C, etc.). The extraction data are shown in Table S1 (in the Supplementary Materials).

### 3.4. Determination of Total Phenolic and Flavonoid Contents

All measurements were performed on 96-well microplates (Nunclon, Nunc; Roskilde, Denmark) using an Infinite Pro 200F microplate reader from Tecan Group Ltd. (Männedorf, Switzerland). The experiments were performed in triplicate.

#### 3.4.1. Total Phenolic Content

The total phenolic content (TPC) was determined spectrophotometrically using the Folin–Ciocalteu method with slight modifications [43]. The Folin–Ciocalteu reagent diluted with water 1:4 (*v*/*v*, 20 μL) and 160 μL of sodium carbonate (75 g/L) was added to each well containing 20 μL of the sample. Methanol was used as a blank. The absorbance of the mixture was read at 680 nm after 20 min. incubation. The results were determined as mg of gallic acid equivalent (GAE) per 1 g of dry extract.

#### 3.4.2. Total Flavonoid Content

The AlCl_3_ method described by Lamaison and Carnet with some modification [15,44] was used to determine the total flavonoid content (TFC) in the sample extracts. First, 20 μL of the sample was mixed with 160 μL of methanol and 20 μL of a solution of 2% (*w*/*v*) AlCl_3_ × 6H_2_O in methanol. The mixture was vigorously shaken and absorbance at 430 nm was read after 30-min incubation. Methanol was used instead of the extract in the blank. All experiments were performed in triplicate. The results were expressed as mg of quercetin (Q) per 1 g of dry weight of the plant material.

### 3.5. Determination of Antioxidant Capacity

DPPH^•^,ABTS^•+^, and metal chelation measurements were performed on 96-well microplates (Nunclon, Nunc; Roskilde, Denmark), and the ORAC measurement was performed on 96-well black microplates (Nunclon, Nunc; Roskilde, Denmark). An Infinite Pro 200F microplate reader from Tecan Group Ltd. (Männedorf, Switzerland) was used in all tests. The experiments were performed in triplicate.

#### 3.5.1. Scavenging Activity of DPPH^•^ Radical

The antioxidant activity was determined with the DPPH^•^ (2,2-diphenyl-1-picrylhydrazyl) method with modifications [43,45]. The tested extracts (20 µL) were applied to 96-well microplates in appropriate concentration ranges. Next, 180 μL of a DPPH^•^ solution, with a concentration of 0.07 mg/mL, was added and incubated for 30 min at 28 °C. Absorbance was measured at 517 nm. The blank contained methanol instead of the sample. The ability to scavenge the DPPH^•^ radical was calculated using the following formula:

$$\%\;Scavenging\;rate=\frac{A_{0}-A_{1}}{A_{0}}*100\%$$

Abbreviations: *A*_0_ is the absorbance of the control (DPPH^•^ solution and solvent instead of the sample); *A*_1_ is the absorbance of the sample.

The results of antioxidant activity were expressed as Trolox equivalent antioxidant capacity (mg of Trolox per 1 g sample) based on their EC_50_ values.

#### 3.5.2. Scavenging Activity of ABTS^•+^ Radical

The antiradical activity was assessed using an improved ABTS^•+^ [2,2-azinobis-(3-ethylbenzothiazoline-6-sulphonic acid)] decolourisation assay, which was described previously [46]. First, quantities of 20 μL of the samples were diluted to various concentrations and mixed with 180-μLaliquots of an ABTS^•+^ solution in 96-well microplates. Absorbance was measured after 6-min incubation at 734 nm. The ABTS radical scavenging activity was expressed as percentage inhibition and calculated using the following equation:

$$\%\;Scavenging\;rate=\frac{A_{0}-A_{1}}{A_{0}}*100\%$$

Abbreviations: *A*_0_ is the absorbance of the control (ABTS^•+^ solution and solvent instead of the sample); *A*_1_ is the absorbance of the sample.

The results of antioxidant activity were expressed as Trolox equivalent antioxidant capacity (TE) (mg of Trolox per 1 g sample) based on their EC_50_ values.

#### 3.5.3. Oxygen Radical Absorbance (ORAC) Assay

The ORAC assay was based on the procedure described by Olech et al. [11]. This method analyses the peroxyl radical scavenging activity of the samples. Free radicals are produced by AAPH [2,2’-azobis(2-amidinopropane) dihydrochloride] and fluorescein is oxidised, losing its fluorescence. All reagents and samples were prepared in 75 mM phosphate buffer (pH 7.4). The working fluorescein solution (10 nM) was freshly prepared by dilution of the stock solution (100 µM) before each analysis. After the addition of AAPH, the plate was shaken for 5 s, then the fluorescence was measured every 90 s for 120 min with an emission and excitation wavelength of λ = 535 and 485 nm, respectively. All fluorescence measurements were made at 37 °C. The oxidative radical absorption capacities of the samples and standards (Trolox) were calculated according to the formula:

AUC = 0.5 × [2 × A_0_ + A_1_ + … + A_n−1_ + A_n_] − A_0_ − A_n_] × Δt,

Abbreviations: A_0_ is the initial fluorescence read at 0 min; A_n_ is the last measurement.

Net AUC = AUC_sample_ − AUC_blank_

The standard curve used different concentrations of Trolox solution as the abscissa and Net AUC as the ordinate [47]. The ORAC values were calculated on the basis of the Area Under the Curve (AUC) results with the data expressed as mM of Trolox equivalents per 1 g of dry extract using the Trolox and the sample calibration curves obtained in each analysis.

#### 3.5.4. Metal-Chelating Activity (CHEL)

The chelating activity of the extracts on Fe^2+^ was determined according to a method that was described previously [11]. First, samples (200 µL) were mixed with 50 µL of a FeCl_2_ solution (0.4 mM) and shaken for 1 min. Next, 100 µL of a ferrozine solution (1 mM) was added and the mixture was shaken vigorously and left standing at room temperature. After incubating for 10 min, absorbance of the mixture was recorded at 562 nm. The blank contained water instead of the sample. Ethylenediaminetetra acetic acid (EDTA) disodium salt (Na_2_EDTA) was used as a positive control. The chelating activity was calculated with the following formula:

(1) $$\%\;Scavenging\;rate=\frac{A_{0}-A_{1}}{A_{0}}*100\%$$

Abbreviations: *A*_0_ is the absorbance of the control; *A*_1_ is the absorbance of the sample.

The results were expressed as EC_50_.

### 3.6. Inhibition of Pro-Oxidant Enzymes

All measurements were performed on 96-well UV microplates using a microplate reader (Epoch 2 Microplate Spectrophotometer, BioTek Instruments, Winooski, VT, USA). The experiments were performed in triplicate.

#### 3.6.1. Inhibition of LOX Activity

Inhibition of 15-lipoxygenase (LOX) was determined as described previously, with some modifications, using soybean 15-LOX [48]. The LOX inhibition was determined spectrophotometrically at 30 °C by measuring the increase in absorbance at λ = 234 nm over a 2-min period. The reaction mixture contained 240 µL of phosphate buffer (1/15 M, pH 7.5), 10 μL of the *Aerva lanata* (L.) Juss. sample, 10 μL of the LOX solution, and 40 μL of 2.5 mM linoleic acid. Ethanol was used as the control instead of the sample. Quercetin was applied as an inhibitor control. All measurements were carried out in triplicate. The mode of inhibition of the enzyme was shown using the Lineweaver–Burk plot.

#### 3.6.2. Inhibition of XO Activity

Inhibition of xanthine oxidase (XO) was determined as described previously with some modifications [49]. The assay mixture was incubated at 30 °C with absorbance (λ = 295 nm) measured spectrophotometrically over a 2-min period. The XO inhibitory activity was expressed as the percentage inhibition of XO in the assay mixture system. The mode of inhibition on the enzyme was shown using the Lineweaver–Burk plot.

### 3.7. Inhibition of Skin Ageing-Related Enzyme Inhibitory Activity

All tests were conducted using 96-well plates (Nunclon, Nunc, Roskilde, Denmark) and the results were estimated with an Infinite Pro 200F Elisa Reader (Tecan Group Ltd., Männedorf, Switzerland). All experiments were performed in triplicate.

#### 3.7.1. Anti-Tyrosinase Activity

Anti-tyrosinase activity was estimated using the method described earlier by Zengin and co-authors [50]. Mushroom tyrosinase (40 µL, 200 U/mL) and *A. lanata* (L.) Juss. samples (20 µL; in different concentrations) or quercetin and tiliroside solutions (20 µL; 25–200 µL/mL and 12.5–100 µL/mL, respectively) were incubated in sodium phosphate buffer (100 µL, pH 6.8) for 10 min at 29 °C. To start the reaction, L-DOPA (40 µL, 0.5 mM) was added. The blank sample had no tyrosinase solution. The change in absorbance after 10-min incubation was measured at 492 nm at 29 °C. Kojic acid (12.5–100 µg/mL) was used as a positive control.

#### 3.7.2. Anti-Elastase Activity

Anti-elastase activity was determined spectrophotometrically according to Chiocchio et al. with some modifications [51,52]. Porcine pancreatic elastase (3.33 mg/mL; 25 µL) and *A. lanata* (L.) Juss. samples (20 µL; in different concentrations) or quercetin and tiliroside solutions (20 µL; 12.5–100 µL/mL) were incubated in Tris-buffer (0.2 mM, pH 8.0) for 10 min at 29 °C. To start the reaction, N-succinyl-Ala-Ala-Ala-p-nitroanilide (2 mM; 125 µL) was added as a substrate. After 15-min incubation, the absorbance was measured at 420 nm. Epigallocatechin gallate (12.5–100 µL/mL) was used as a positive control.

#### 3.7.3. Anti-Collagenase Activity

Anti-collagenase activity was determined using N-[3-(2-furyl) acryloyl]-Leu-Gly-Pro-Ala (FALGPA) as a substrate, and activity was measured according to Mandrone et al. with some modifications [52,53] Collagenase from *Clostridium histolyticum* (20 mU) dissolved in Tricine buffer (pH 7.5, 0.05 M, containing 0.4 M sodium chloride and 0.01 M calcium chloride) and *A. lanata* samples (20 µL; in different concentrations) or quercetin and tiliroside solutions (20 µL; 12.25–100 µL/mL), were incubated for 10 min at 29 °C. To start the reaction, FALGPA (1 mM) was added. After 15-min incubation at 29 °C, the absorbance was measured at 340 nm. Epigallocatechin gallate (12.25–100 µL/mL) was used as a positive control.

#### 3.7.4. Anti-Hyaluronidase Activity

The assay was performed following the method suggested by the Sigma protocol with slight modifications [54]. The assay medium containing hyaluronidase (10 µL of 4 U/mL), sodium phosphate buffer (100 µL of 200 mM, pH 7, 37 °C), sodium chloride (77 mM), Bovine Serum Albumin (0.01%), and different concentrations of the sample solution (20 µL) was incubated at 37 °C for 10 min. Next, the reaction was initiated by the addition of the substrate in the form of a hyaluronic acid solution (100 µL of 0.03% in 300 mM sodium phosphate, pH 5.35) and incubated at 37 °C for 45 min. The undigested hyaluronic acid was precipitated with an acid albumin solution (1 mL of 0.1% BSA in 24 mM sodium acetate and 79 mM acetic acid, pH 3.75). After leaving the mixture at room temperature for 10 min, the absorbance of the reaction mixture was measured at 600 nm using a spectrophotometer. All solutions were prepared fresh before the enzyme assay was performed. The absorbance in the absence of the enzyme was used as a control value for maximum inhibition. Epigallocatechin gallate was used as the positive control in this assay.

### 3.8. LC-ESI-MS/MS Analysis

Qualitative and quantitative analyses of polyphenolic compounds in the samples were performed using high-performance liquid chromatography coupled with triple quadrupole tandem mass spectrometry (LC-MS/MS). The method for simultaneous analysis of all the polyphenol groups studied was based on previous experiments, with some modifications [11,55,56]. Separations were carried out using an Agilent 1200 Series LC system (Agilent Technologies, Santa Clara, CA, USA) and a 4.6 × 150 mm Agilent Eclipse XDB-C18 column (5 µm; Agilent Technologies, USA). The mobile phase consisted of water containing 0.1% HCOOH (solvent A) and acetonitrile containing 0.1% HCOOH (solvent B). The flow rate was 350 µL min^−1^, and the mobile phase gradient was programmed as follows: 0–1.5 min, 13% B; 2–4.5 min, 20% B; 5–8 min, 25% B; 9–11 min, 33% B; 13–16 min, 60% B; 18–21 min, 80% B; 23–28 min, 13% B. The injection volume and column temperature were 3 µL and 25 °C, respectively. The LC system was connected to a 3200 QTRAP Mass spectrometer (Sciex, Redwood City, CA, USA) equipped with an electrospray ionisation source (ESI) and working in the multiple reaction monitoring (MRM) scan mode. Nitrogen was used as a curtain and collision gas. ESI worked in the negative ion mode in the following conditions: capillary temperature of 500 °C, curtain gas at 30 psi (pound-force per square inch), nebuliser gas at 55 psi, and negative ionisation mode source voltage of −4500 V. For each analyte, the optimum parameters of the Multiple Reaction Mode (MRM) were determined in the infusion mode. The optimised instrument settings for product ions of each compound are shown in Table S2 (in the Supplementary Materials). Analyst 1.5 software (Sciex, Redwood City, CA, USA) was used for data acquisition and processing. The analytes were identified by comparing retention times and MRM transitions with the parameters from corresponding standards tested in the same conditions. The compounds were quantified on the basis of peak areas of the most intense MRM transitions using the results from calibration curves generated for the corresponding standards.

The LOD (limit of detection) and LOQ (limit of quantification) values were established at a signal-to-noise ratio of 5:1 and 10:1, respectively, based on the results obtained for independent replicates of standard solutions (Table S3 in the Supplementary Materials). The samples were filtered through a hydrophilic polytetrafluoroethylene (PTFE) 0.20 μm membrane (Merck, Darmstadt, Germany) syringe filter prior to LC injection.

### 3.9. Statistical Analysis

All assays were conducted in triplicate. The results were expressed as mean values with the standard deviation of independent measurements. A one-way ANOVA test followed by Tukey’s post hoc test was used for statistical analysis of the differences between the data. Significance was assumed at *p* < 0.05 and *p* < 0.001. All calculations were performed using Microsoft Excel. Three-dimensional surface charts, k-means clusters, and Pearson’s correlation coefficients (*r*) between the extraction conditions, components, and activities of the analysed extracts were obtained in STATISTICA 10.0 (StatSoft Poland, Cracow, Poland).

## 4. Summary and Conclusions

Natural plant products, especially those that are rich in polyphenols, are currently very popular due to the wide range of their pharmacological activity, health effects, and skin photoageing protection. Thus, it is very important to identify new sources of biologically active phenolic compounds. *Aerva lanata* (L.) Juss. (AL) herb is one of the key ingredients in some traditional ayurvedic compositions and is used in the treatment of diseases related to oxidative stress. Our previous study was the first to show AL as a rich source of free and bound phenolics [9].

To the best of our knowledge, this work is the first report on the optimisation of the accelerated solvent extraction of *A. lanata* (L.) Juss. herb extracts showing favourable conditions for effective extraction of phenolic compounds, i.e., flavonoids and phenolic acids, from this plant material. ASE extraction at high temperature was proven to release high amounts of biologically active polyphenols from this plant material.

The use of water–ethanol as a solvent in ASE considerably increased the extraction productivity as compared with pure ethanol and water. For the first time, a simple and efficient ASE method and set of conditions were developed to obtain polyphenolic-rich AL extracts with high antioxidant and anti-inflammatory activity. The optimum extraction conditions established, i.e., the 50% ethanol concentration and 180 °C temperature, yielded the highest content of polyphenols, and the 80% ethanol concentration and 180 °C temperature ensured the highest content of flavonoids. In both cases, the influence of high temperature was the most important factor.

Additionally, both extracts obtained in the selected conditions showed the highest antioxidant activity in the DPPH^•^, ABTS^•+^, ORAC, and XO in vitro tests. In addition, all tested extracts were shown to inhibit lipoxygenase and xanthine oxidase. The highest LOX inhibitory potential was exhibited by the water extracts and the 50% ethanol extract at 180 °C. The extracts showed different modes of enzyme inhibition. Only the 50% ethanol extract at 60 °C showed a competitive mode of LOX inhibition. This may be related to the large amount of tiliroside in this extract. Additionally, low correlations with the content of phenolic compounds and LOX inhibition ability of the extracts were observed. This may mean that, in addition to phenolic compounds, other molecules contribute to anti-inflammatory activities. The highest XO inhibitory potential was shown by the 80% ethanol extract at 180 °C. In this case, the high content of quercetin had a considerable effect on the enzyme activity.

The present research is the first report on the detailed phenolic profile in modern green ASE extracts from *A. lanata* herb. The LC-ESI-MS/MS-MRM analysis revealed the presence of 10 phenolic acids and 11 flavonoids. It was observed that the AL extracts contained large amounts of flavonoids (mainly tiliroside in extracts obtained at lower temperatures and quercetin in extracts obtained at higher temperatures). It is worth noting that our study is the first report on the high content of tiliroside in AL herb. Since tiliroside is known for its high anti-inflammatory activity, its presence in AL extracts is desirable from a practical point of view, taking into account their potential use in cosmetic preparations and pharmaceuticals. Moreover, constituents (*p*-hydroxybenzoic, caffeic, *p*-coumaric, protocatechuic acids, tiliroside, and quercetin) that may be involved in the high ability to inhibit enzymes and act as radical scavengers were indicated.

This phytochemical and pharmacological data may be of great importance for the effective use of AL as a source of phenolic compounds for potential commercial applications.

The results of the present study clearly reveal the antioxidant and anti-inflammatory activity of *A. lanata* (L.) Juss. Due to the determined rich polyphenolic composition and biological properties, especially the high antiradical, antioxidant, and anti-inflammatory activity of the selected ASE extracts from *A. lanata* (L.) Juss. herb, the plant and its extracts have a pro-health effect.

Our study also expanded the knowledge about the anti-ageing potential of AL herb. Additionally, the results showed that the ASE extracts have promising anti-photoageing potential, especially anti-tyrosinase, anti-elastase, anti-collagenase, and anti-hyaluronidase activities, and proved the impact of AL polyphenols (especially tiliroside and quercetin) on the activity of these enzymes. Further, more detailed investigations are, however, needed to confirm these findings before the application of AL in the production of cosmetics.

## Figures and Tables

**Table 1:** Efficiency of extraction (EEx), total phenolic content (TPC), total flavonoid content (TFC), and antioxidant activity assessed with the DPPH^•^ and ABTS^•+^ methods in extracts obtained from *Aerva lanata* (L.) Juss. herb. Explanations: EEx—calculated per dry extract obtained from 1 g of raw material; TPC—calculated as mg of GA per 1 g DE (of dry extract); TFC—calculated as mg of Q per 1 g DE; DPPH^•^ and ABTS^•+^—calculated as mg of Trolox per 1 g DE. Abbreviations: W—water extracts; E—ethanol and ethanol–water extracts. The first number in the sample symbols means the percent ethanol concentration in the extraction solvent, and the extraction temperature is shown in the brackets.

Sample	EEx (%)	TPC	TFC	DPPH^•^	ABTS^•+^
E100 (60 °C)	3.56	15.83 ^a^ ± 0.25	5.53 ^ab^ ± 0.15	10.07 ^a^ ± 0.23	9.90 ^a^ ± 0.01
E100 (80 °C)	5.20	18.48 ^ab^ ± 0.56	8.32 ^b^ ± 0.35	9.17 ^a^ ± 0.07	12.77 ^ac^ ± 0.00
E100 (100 °C)	6.69	25.03 ^c^ ± 0.14	8.52 ^b^ ± 0.52	10.28 ^a^ ± 0.46	12.31 ^ac^ ± 0.21
E100 (180 °C)	12.20	45.20 ^d^ ± 0.25	11.54 ^c^ ± 0.28	72.48 ^b^ ± 0.91	98.94 ^b^ ± 1.75
E80 (60 °C)	7.63	15.91 ^a^ ± 0.09	6.53 ^ab^ ± 0.06	19.86 ^cd^ ± 0.01	11.60 ^ac^ ± 0.10
E80 (80 °C)	8.07	14.62 ^a^ ± 0.45	7.46 ^ab^ ± 0.09	18.02 ^ci^ ± 0.09	14.37 ^c^ ± 0.28
E80 (100 °C)	10.00	33.58 ^e^ ± 0.27	9.43 ^bc^ ± 0.85	48.43 ^f^ ± 1.60	50.22 ^e^ ± 0.36
E80 (180 °C)	19.31	46.24 ^d^ ± 0.91	12.89 ^c^ ± 0.92	119.85 ^g^ ± 1.95	107.58 ^f^ ± 1.32
E50 (60 °C)	12.32	28.78 ^f^ ± 0.56	6.79 ^ab^ ± 0.18	24.84 ^e^ ± 0.52	34.72 ^g^ ± 0.80
E50 (80 °C)	10.15	31.92 ^e^ ± 0.12	6.92 ^ab^ ± 0.08	23.28 ^e^ ± 0.00	36.11 ^g^ ± 1.06
E50 (100 °C)	10.58	50.18 ^g^ ± 1.05	7.10 ^ab^ ± 0.24	24.28 ^e^ ± 1.18	33.61 ^g^ ± 0.83
E50 (180 °C)	31.22	53.43 ^h^ ± 2.05	11.70 ^c^ ± 0.30	82.63 ^h^ ± 0.90	81.16 ^h^ ± 3.81
W (60 °C)	15.23	19.69 ^b^ ± 0.24	4.10 ^a^ ± 0.21	15.22 ^i^ ± 0.71	38.61 ^d^ ± 0.48
W (80 °C)	16.58	22.88 ^c^ ± 1.00	3.66 ^a^ ± 0.25	16.27 ^i^ ± 0.67	42.16 ^j^ ± 0.34
W (100 °C)	17.50	20.89 ^b^ ± 0.23	5.35 ^ab^ ± 0.19	21.71 ^de^ ± 0.94	34.57 ^g^ ± 1.88
W (180 °C)	38.24	16.87 ^a^ ± 0.31	7.26 ^b^ ± 0.27	79.94 ^h^ ± 1.12	88.12 ^i^ ± 1.26

Values are presented as mean ± standard deviation (*n* = 3) and evaluated by one-way ANOVA test (post-test: Tukey). Different superscript letters (^a–j^) in the same column denote significant differences at *p* < 0.05.

**Table 2:** Pearson’s correlation coefficients (*r*) between different extraction conditions, efficiency of extraction (EEx), total phenolic content (TPC), total flavonoid content (TFC), and antioxidant activity (DPPH^•^ and ABTS^•+^ methods) in *Aerva lanata* (L.) Juss. herb extracts.

	Temperature	Solvent Concentration	TPC	TFC
EEx	0.761	−0.597	-	-
TPC	0.694	0.288	-	-
TFC	0.835	0.402	-	-
DPPH^•^	0.935	0.033	0.720	0.880
ABTS^•+^	0.949	−0.076	0.722	0.816

**Table 3:** Composition of clusters determined by cluster analysis (k-means method). Abbreviations: see Table 1.

Cluster 1	Cluster 2	Cluster 3	Cluster 4
E80 (100 °C)	E80 (60 °C)	E100 (180 °C)	E100 (60 °C)
E50 (60 °C)	E80 (80 °C)	E80 (180 °C)	E100 (80 °C)
E50 (80 °C)	W (60 °C)	E50 (180 °C)	E100 (100 °C)
E50 (100 °C)	W (80 °C)	-	-
W (180 °C)	W (100 °C)	-	-

**Table 4:** Content of phenolic acids and flavonoids in some extracts obtained from the herb of *Aerva lanata* (L.) Juss. Mean values of three tests (*n* = 3) with standard deviation (±SD). Abbreviations: <LOQ—limit of quantitation; N/D—not detected; for the other abbreviations, see Table 1.

Compound/Sample	E100 (60 °C)	E100 (180 °C)	E80 (60 °C)	E80 (180 °C)	E50 (60 °C)	E50 (180 °C)	W (60 °C)	W (180 °C)
Phenolic acids (μg/g of dry extract)								
Salicylic	<LOQ	<LOQ	<LOQ	<LOQ	<LOQ	<LOQ	<LOQ	<LOQ
4-hydroxybenzoic	340.5 ± 0.2	35.1 ± 1.2	260.0 ± 4.4	<LOQ	317.0 ± 1.8	9.1 ± 0.2	521.0 ± 3.8	<LOQ
Gentistic	<LOQ	<LOQ	<LOQ	<LOQ	N/D	<LOQ	N/D	<LOQ
Protocatechuic	92.1 ± 0.8	128.5 ± 1.7	86.5 ± 4.1	105.5 ± 2.0	N/D	79.2 ± 3.5	N/D	81.4 ± 3.9
*p*-coumaric	97.8 ± 4.6	117.0 ± 0.0	135.0 ± 2.1	86.3 ± 2.4	157.5 ± 2.2	111.5 ± 1.9	N/D	144.5 ± 0.5
Vanillic	25.3 ± 0.8	<LOQ	<LOQ	N/D	N/D	N/D	<LOQ	N/D
Gallic	<LOQ	N/D	<LOQ	<LOQ	<LOQ	<LOQ	<LOQ	<LOQ
Caffeic	N/D	N/D	<LOQ	<LOQ	N/D	<LOQ	N/D	N/D
Syringic	27.1 ± 1.2	47.3 ± 0.4	77.9 ± 1.8	<LOQ	N/D	<LOQ	N/D	37.9 ± 1.8
Ferulic	N/D	N/D	N/D	N/D	N/D	N/D	N/D	<LOQ
Flavonoids (µg/g of dry extract)								
Astragalin	132.5 ± 1.6	297.0 ± 0.5	288.0 ± 2.5	130.5 ± 4.9	109.5 ± 0.6	82.0 ± 3.9	N/D	N/D
Tiliroside	514.0 ± 0.7	591.5 ± 0.6	1565.0 ± 0.5	205.5 ± 1.0	1237.5 ± 4.9	193.0 ± 1.5	<LOQ	<LOQ
Narcissin	46.7 ± 2.2	80.4 ± 1.1	118.0 ± 3.6	59.1 ± 2.7	98.2 ± 4.8	54.8 ± 2.5	30.4 ± 0.3	26.2 ± 0.9
Rutin	<LOQ	<LOQ	<LOQ	<LOQ	<LOQ	<LOQ	<LOQ	<LOQ
Quercetin	N/D	248.5 ± 0.9	29.1 ± 1.3	296.5 ± 1.1	N/D	316.0 ± 0.9	N/D	2.7 ± 0.5
Kaempferol	<LOQ	37.2 ± 0.6	21.5 ± 0.6	27.2 ± 0.0	<LOQ	36.3 ± 0.4	<LOQ	<LOQ
Isorhamnetin	<LOQ	55.8 ± 0.4	12.8 ± 0.3	51.2 ± 2.1	N/D	61.7 ± 0.3	N/D	<LOQ
Apigenin	<LOQ	14.3 ± 0.4	<LOQ	5.9 ± 0.2	<LOQ	<LOQ	<LOQ	<LOQ
Prunetin	2.1 ± 0.1	2.4 ± 0.1	3.6 ± 0.2	3.5 ± 0.1	1.8 ± 0.0	1.9 ± 0.0	1.7 ± 0.0	1.7 ± 0.1
Luteolin	N/D	<LOQ	N/D	N/D	N/D	N/D	N/D	<LOQ
Rhamnetin	<LOQ	<LOQ	<LOQ	<LOQ	<LOQ	<LOQ	<LOQ	<LOQ

**Table 5:** Lipoxygenase inhibitory (LOX) and xanthine oxidase (XO), chelating power (CHEL), and oxygen radical absorbance capacity (ORAC) in extracts obtained from the herb of *Aerva lanata* (L.) Juss. Mean values of three tests with standard deviation (±SD). Explanations: ORAC—calculated as mM Trolox per g of dry extract; LOX, XO, and CHEL—calculated as EC_50_ (mg per mL). NA—no activity.

	ORAC	CHEL	LOX	XO
E100 (60 °C)	0.36 ^a^ ± 0.06	1.58 ^a^ ± 0.17	3.73 ^a^ ± 0.03	3.72 ^ab^ ± 0.17
E100 (180 °C)	1.85 ^b^ ± 0.19	5.30 ^b^ ± 0.70	3.65 ^a^ ± 0.20	3.65 ^a^ ± 0.14
E80 (60 °C)	0.93 ^c^ ± 0.20	3.42 ^c^ ± 0.06	3.51 ^a^ ± 0.17	2.67 ^c^ ± 0.11
E80 (180 °C)	2.35 ^d^ ± 0.18	5.10 ^b^ ± 0.01	2.42 ^b^ ± 0.12	1.28 ^d^ ± 0.00
E50 (60 °C)	0.90 ^c^ ± 0.30	2.59 ^d^ ± 0.18	2.85 ^d^ ± 0.15	2.82 ^ace^ ± 0.10
E50 (180 °C)	3.84 ^e^ ± 0.06	3.70 ^c^ ± 0.08	1.24 ^c^ ± 0.04	2.07 ^f^ ± 0.11
W (60 °C)	0.81 ^c^ ± 0.09	NA ^e^	1.14 ^c^ ± 0.01	3.48 ^b^ ± 0.02
W (180 °C)	2.32 ^d^ ± 0.20	NA ^e^	1.15 ^c^ ± 0.12	3.02 ^ae^ ± 0.00

Values are presented as mean ± standard deviation (*n* = 3) and evaluated by one-way ANOVA test (post-test: Tukey). Different superscript letters (^a-–f^) in the same column denote significant differences at *p* < 0.05.

**Table 6:** Pearson’s correlation coefficients (*r*) showing a relationship between the content of phenolic acids and flavonoids determined with the LC/MS method and the extraction conditions and biological activities.

Compound	Temperature	Solvent Concentration	DPPH^•^	ABTS^•+^	ORAC	LOX	XO	CHEL
Phenolic acids								
4-hydroxybenzoic	−0.928	−0.187	−0.892	−0.819	−0.810	0.105	0.621	−0.535
Protocatechuic	0.407	0.636	0.369	0.202	0.214	0.412	−0.302	0.390
*p*-Coumaric	0.188	0.274	0.146	0.050	0.170	0.347	−0.168	0.273
Vanillic	−0.374	0.426	−0.427	−0.500	−0.462	0.446	0.337	−0.221
Syringic	−0.072	0.301	−0.203	−0.230	−0.241	0.495	0.283	0.780
Flavonoids								
Astragalin	0.051	0.861	0.017	0.008	−0.129	0.846	−0.105	0.782
Tiliroside	−0.536	0.465	−0.473	−0.565	−0.454	0.693	0.053	0.308
Narcissin	−0.377	0.646	−0.307	−0.410	−0.311	0.764	−0.113	0.549
Quercetin	0.760	0.371	0.788	0.743	0.774	−0.064	−0.731	0.790
Kaempferol	0.620	0.500	0.594	0.553	0.643	0.155	−0.584	0.861
Isorhamnetin	0.737	0.421	0.730	0.704	0.745	0.010	−0.660	0.824
Apigenin	0.521	0.507	0.456	0.600	0.159	0.402	−0.132	0.682
Prunetin	0.052	0.568	0.259	0.046	−0.052	0.480	−0.574	0.610
Luteolin	0.378	0.426	0.193	0.409	0.064	0.418	0.198	0.506

**Table 7:** Anti-tyrosinase, anti-collagenase, anti-elastase, and anti-hyaluronidase activities expressed as EC_50_ of the selected extracts of the herb of *A. lanata* (L.) Juss., quercetin, tiliroside, kojic acid, and EGCG. Abbreviations: EGCG—epigallocatechin gallate; NT—not tested; NA—no activity.

Sample	EC_50_ (µg/mL)			
	Tyrosinase	Collagenase	Elastase	Hyaluronidase
E100(60 °C)	46.48 ^a^ ± 2.93	598.23 ^a^ ± 2.65	NA^a^	160.84 ^a^ ± 2.22
E100(180 °C)	52.19 ^b^ ± 2.84	78.47 ^b^ ± 0.29	57.26 ^b^ ± 0.30	126.81 ^b^ ± 3.26
E80(60 °C)	43.32 ^c^ ± 0.57	129.44 ^c^ ± 0.49	95.25 ^c^ ± 1.26	117.54 ^c^ ± 4.82
E80(180 °C)	60.10 ^d^ ± 0.44	59.73 ^d^ ± 0.31	22.54 ^d^ ± 1.86	215.41 ^d^ ± 4.97
E50(60 °C)	46.08 ^e^ ± 0.29	158.08 ^e^ ± 3.61	153.82 ^e^ ± 2.88	131.22 ^e^ ± 0.62
E50(180 °C)	58.56 ^f^ ± 0.37	21.76 ^f^ ± 1.27	35.81 ^f^ ± 0.81	237.54 ^f^ ± 7.88
W(60 °C)	155.97 ^g^ ± 1.34	663.07 ^g^ ± 14.08	NA^a^	156.60 ^g^ ± 1.83
W(180 °C)	124.62 ^h^ ± 1.14	134.32 ^h^ ± 3.20	166.23 ^g^ ± 5.42	135.29 ^h^ ± 5.19
Quercetin	91.81 ^i^ ± 0.56	59.06 ^i^ ± 1.08	13.06 ^h^ ± 0.18	117.74 ^c^ ± 1.35
Tiliroside	24.92 ^j^ ± 0.29	69.53 ^j^ ± 0.37	22.08 ^d^ ± 0.17	128.67 ^i^ ± 1.28
Kojic acid	28.42 ^k^ ± 0.11	NT	NT	NT
EGCG	NT	55.31 ^k^ ± 0.64	27.29 ^i^ ± 0.25	94.86 ^j^ ± 0.52

Values are presented as mean ± standard deviation (*n* = 3) and evaluated by one-way ANOVA test (post-test: Tukey). Different superscript letters (^a–k^) in the same column denote significant differences at *p* < 0.05.

**Table 8:** Pearson’s correlation coefficients (*r*) between the skin ageing-related enzymes and concentrations of secondary metabolites in AL extracts. Abbreviations: TYR—tyrosinase; COL—collagenase; ELA—elastase; HYA—hyaluronidase activity.

	TYR	COL	ELA	HYA
Sum of phenolic acids	−0.030	0.696	0.492	−0.610
Sum of flavonoid aglycones	−0.358	−0.623	−0.939	0.647
Sum of flavonoid glycosides	−0.731	−0.403	−0.197	−0.205

## Data Availability

Not applicable.

## Supplementary Materials

Table S1: Conditions of accelerated solvent extraction; Table S2: LC-ESI-MS/MS analytical results of phenolic acids and flavonoids investigated in the samples. The compounds were confirmed by comparison with authentic standards; Table S3: Limit of detection (LOD), limit of quantification (LOQ), and calibration curve parameters for phenolic acids and flavonoids.

## Sample Availability

Samples of the compounds are available from the authors.
