# Supplementary material for: LC-ESI-MS/MS Polyphenolic Profile and In Vitro Study of Cosmetic Potential of Aerva lanata (L.) Juss. Herb Extracts
Source: Molecules. 2022 Feb 13;27(4):1259. doi: 10.3390/molecules27041259 (PMC8879310; doi:10.3390/molecules27041259)
Supplement: Supplementary file 1 [file molecules-27-01259-s001.zip › molecules-1564916-supplementary.pdf]

Supplementary Materials

LC-ESI-MS/MS polyphenolic profile and *in vitro* study of cosmetic potential of *Aerva lanata* (L.) Juss. herb extracts

Aleksandra Pieczykolan[1], Wioleta Pietrzak[1], Katarzyna Dos Santos Szewczyk[1], Urszula Gawlik-Dziki [2] and Renata Nowak[1,*]

[1]Department of Pharmaceutical Botany, Medical University, 1 Chodźki Street, 20-093 Lublin, Poland; aleksandraoleszek@umlub.pl (A.P.); wioleta.pietrzak@umlub.pl (W.P.); k.szewczyk@umlub.pl (K.D.S.S.)

[2]Department of Biochemistry and Food Chemistry, University of Life Sciences, 8 Skromna Street, 20-704 Lublin, Poland; urszula.gawlik@up.lublin.pl; (U.G.-D.)

*Correspondence: renata.nowak@umlub.pl (R.N.); Tel.: +48 81 448 70 62

Abstract: The aim of the present study was to investigate the phenolic composition and the biological properties of different *Aerva lanata* (L). Juss. herb extracts obtained with the use of accelerated solvent extraction (ASE), i.e. a green, ecological method, for cosmetic purposes. All samples exhibited high DPPH$^{\bullet}$ (9.17–119.85 mg TE/g) and ABTS$^{\bullet+}$ (9.90–107.58 mg TE/g) scavenging activity. The extracts exhibited considerable anti-lipoxygenase (EC_{50} between 1.14 mg/mL and 3.73 mg/mL) and anti-xanthine oxidase (EC_{50} between 1.28 mg/mL and 3.72 mg/mL) activities, moderate chelating activity (EC_{50} between 1.58 mg/mL and 5.30 mg/mL), and high antioxidant potential in the ORAC assay (0.36–3.84 mM TE/g). Changes in the polyphenol profile of the analysed samples depending on the solvent and temperature used for the extraction were determined with the liquid chromatography/electrospray mass spectrometry (LC-ESI-MS/MS) method. Twenty-one phenolic compounds, including flavonoids and phenolic acids, were detected and quantified. It was shown that tiliroside was one of the main phenolic metabolites in the *A. lanata* (L.) Juss herb., which may suggest that this compound may be largely responsible for the observed anti-inflammatory activity of the extracts. Also, the studied extracts exhibited promising skin-related (anti-tyrosinase, anti-elastase, anti-collagenase, and anti-hyaluronidase) activity. This study has shown that *Aerva lanata* (L.) Juss. contains high amounts of phenolic compounds, including tiliroside, and has good skin-related activities. Therefore, the plant may be interesting as a novel source of bioactive agents for cosmetic industries.

Keywords: *Aerva* extract; polpala; ASE; LC-ESI-MS/MS; MRM; phenolic compounds; tiliroside; ORAC; cosmetic potential

Table S1: Conditions of accelerated solvent extraction

Sample name	Type of solvent Ethanol/water	Concentration of ethanol [%]	Temperature [°C]
E100 (60°C)	Ethanol	100	60
E100 (80°C)	Ethanol	100	80
E100 (100°C)	Ethanol	100	100
E100 (180°C)	Ethanol	100	180
E80 (60°C)	Ethanol	80	60

E80 (80°C)	Ethanol	80	80
E80 (100°C)	Ethanol	80	100
E80 (180°C)	Ethanol	80	180
E50 (60°C)	Ethanol	50	60
E50 (80°C)	Ethanol	50	80
E50 (100°C)	Ethanol	50	100
E50 (180°C)	Ethanol	50	180
W (60°C)	Water	0	60
W (80°C)	Water	0	80
W (100°C)	Water	0	100
W (180°C)	Water	0	180

Table S2: LC-ESI-MS/MS analytical results of phenolic acids and flavonoids investigated in samples. Compounds were confirmed by comparison with authentic standards

**Compound**	**Retention time [min]**	**[M-H]$^-$ [m/z]**	**Fragment ions [m/z]**	**Collision energy [eV]**
Phenolic acids				
Gallic acid	5.00	168.7	78.9<br>124.9	-36<br>-14
Protocatechuic acid	5.85	152.9	80.9<br>107.8	-26<br>-38
Caffeic acid	6.70	178.7	88.9<br>134.9	-46<br>-16
Syringic acid	7.14	196.9	122.8<br>181.9	-24<br>-12
4-hydroxybenzoic acid	7.27	136.8	92.9	-18
Vanillic acid	7.46	166.8	107.9<br>123	-18<br>-12
Gentisic acid	7.72	352.9	80<br>96.9	-110<br>-52
p-Coumaric acid	9.28	162.8	93<br>119	-44<br>-14
Ferulic acid	9.84	192.8	133.9<br>177.9	-16<br>-12
Salicylic acid	14.16	136.9	75<br>93	-48<br>-16
Flavonoids				
Apigenin	18.64	268.8	117<br>106.8	-44<br>-34
Prunetin	21.98	282.2	267.7<br>238.7	-20<br>-16
Kaempferol	18.85	284.7	116.8<br>93	-46<br>-52

Luteolin	17.85	284.7	132.9	-38
			150.9	-26
Quercetin	17.94	300.7	150.9	-26
			178.8	-20
Rhamnetin	20.10	314.7	165	-24
			120.9	-36
Isorhamnetin	18.99	314.7	299.7	-20
			150.9	-30
Astragalin	14.66	446.7	226.8	-54
			254.8	-40
Rutin	11.99	608.7	299.6	-46
			270.9	-60
Tiliroside	17.39	592.8	284.8	-38
			254.7	-56
Narcissin	13.52	622.8	314.9	-40
			298.8	-52

Table S3:Limits of detection (LOD), limits of quantification (LOQ), and calibration curve parameters for phenolic acids and flavonoids

**Compound**	**LOD [ng/mL]**	**LOQ [ng/mL]**	**R^2**	**Linearity range [ng/mL]**
		Phenolic acids		
Gallic acid	47.5	95	0.9980	190-3300
Protocatechuic acid	17	34	0.9992	68-3470
Caffeic acid	40	160	0.9990	320-3500
Syringic acid	166	666	0.9985	666-11100
4-hydroxybenzoic acid	17	34.7	0.9984	104.1-3470
Vanillic acid	125	250	0.9987	500-33000
Gentisic acid	1.6	3.3	0.9991	3.3-330
p-Coumaric acid	7.3	18.1	0.9991	36.2-1820
Ferulic acid	17.4	34.7	0.9991	69.4-11600
Salicylic acid	8.3	16.5	0.9985	16.5-1650
		Flavonoids		
Apigenin	15	30	0.9979	89-4470

Prunetin	10	20	0.9983	150-20000
Kaempferol	33	66	0.9987	89-4470
Luteolin	5	15	0.9982	330-1500
Quercetin	66	120	0.9987	120-6600
Rhamnetin	120	240	0.9988	240-4400
Isorhamnetin	300	500	0.9991	1000-5000
Astragalin	100.0	200.0	0.9978	1200-24000
Rutin	120.0	300.0	0.9985	2000-25000
Tiliroside	100	250	0.9981	1000-25000
Narcissin	50	100	0.9983	200-25000
